# 

**Published:** 1888-04

**Authors:** 


					﻿JkFZRJL, 1888.
OLD SERIES
VoLXXV
NEW .SERI ES
Vol. V.--No 2\f
4* 18550

HedigIMS®
4» JmmNsiScS?^
tSg|!pigB&


v W.F. WESTMOREL AND. M D
H.V.M.MILLER.M.D.LL.D4
'XbUSHEDBY JAMES P.HARRISQN&cd
&	$ST LANTA .GA. M
SUBSCRIPTIONS S2.5O A YEAR.
				

